# Prognostic impact of malnutrition on cardiovascular events in coronary artery disease patients with myocardial damage

**DOI:** 10.1186/s12872-021-02296-9

**Published:** 2021-10-06

**Authors:** Ryo Arikawa, Daisuke Kanda, Yoshiyuki Ikeda, Akihiro Tokushige, Takeshi Sonoda, Kazuhiro Anzaki, Mitsuru Ohishi

**Affiliations:** Department of Cardiovascular Medicine and Hypertension, Graduate School of Medical and Dental Sciences, Kagoshima University, 8-35-1 Sakuragaoka, Kagoshima City, Kagoshima 890-8520 Japan

**Keywords:** Malnutrition, Coronary artery disease, Myocardial damage, Percutaneous coronary intervention, Hemodialysis

## Abstract

**Background:**

Stable coronary artery disease (CAD) patients with myocardial damage have a poor prognosis compared to those without myocardial damage. Recently, malnutrition has been reported to affect the prognosis of cardiovascular diseases. However, the effects of malnutrition on prognosis of CAD patients with myocardial damage remains uncertain. We investigated the effects of malnutrition on prognosis of CAD patients with myocardial damage who received percutaneous coronary intervention (PCI).

**Methods:**

Subjects comprised 241 stable CAD patients with myocardial damage due to myocardial ischemia or infraction. Patients underwent successful revascularization for the culprit lesion by PCI using second-generation drug-eluting stents and intravascular ultrasound. The geriatric nutritional risk index (GNRI), which is widely used as a simple method for screening nutritional status using body mass index and serum albumin, was used to assess nutritional status. Associations between major cardiovascular and cerebrovascular events (MACCE) and patient characteristics were assessed.

**Results:**

Mean GNRI was 100 ± 13, and there were 55 malnourished patients (23%; GNRI < 92) and 186 non-malnourished patients (77%). MACCE occurred within 3 years after PCI in 42 cases (17%), including 34 deaths (14%), and the malnourished group showed a higher rate of MACCE (38%) compared with the non-malnourished group (11%, *p* < 0.001). Univariate Cox proportional hazards analyses showed that MACCE was associated with age [hazard ratio (HR), 1.04; 95% confidence interval (CI), 1.04–1.07; *p* = 0.004], prior heart failure (HR 2.35; 95% CI 1.10–5.01; *p* = 0.027), high-sensitivity C-reactive protein (HR 1.08; 95% CI 1.03–1.11; *p* < 0.001), hemodialysis (HR 2.63; 95% CI 1.51–4.58; *p* < 0.001) and malnutrition (HR 3.69; 95% CI 2.11–6.42; *p* < 0.001). Multivariate Cox proportional hazards analysis revealed hemodialysis (HR 2.17; 95% CI 1.19–3.93; *p* = 0.011) and malnutrition (HR 2.30; 95% CI 1.13–4.67; *p* = 0.020) as significantly associated with MACCE. Furthermore, Cox proportional hazards models using malnutrition and hemodialysis revealed that patients with malnutrition and hemodialysis were at greater risk of MACCE after PCI than patients with neither malnutrition nor hemodialysis (HR 6.91; 95% CI 3.29–14.54; *p* < 0.001).

**Conclusions:**

In CAD patients with myocardial damage, malnutrition (GNRI < 92) represents an independent risk factor for MACCE. Assessment of nutritional status may help stratify the risk of cardiovascular events and encourage improvements in nutritional status.

## Background

Cardiovascular disease is the leading cause of mortality and disability around the world [1]. Despite recent clinical advances in percutaneous coronary intervention (PCI) and medical therapy [2], coronary artery disease (CAD) remains a major issue worldwide [1]. Assessment of risk stratification for mortality and cardiovascular events in patients with CAD is therefore crucial when making medical decisions [3, 4].

Malnutrition is common among hospitalized patients and has been reported to be associated with worsened prognosis among patients with chronic diseases such as cancer [5] and renal failure [6]. Various tools are available for the evaluation of nutritional status, and the geriatric nutritional risk index (GNRI) is widely used as a simple method for screening nutritional status using body mass index (BMI) and serum albumin [7]. The GNRI was a tool created to study and predict nutrition-related complications in elderly patients and was initially proposed for sub-acute care setting [7]. Recent studies have demonstrated that GNRI is associated with worsened prognosis in heart failure patients [8], and with mortality in patients with chronic life-threatening ischemia [9]. GNRI could thus be important for risk stratification even in patients with cardiovascular disease.

Stable CAD patients with myocardial damage display poor prognosis compared to those without myocardial damage [10]. However, the impact of malnutrition on the prognosis of CAD patients with myocardial damage remains unclear. The present study aimed to evaluate the prognostic value of nutritional status using GNRI in stable CAD patients with myocardial damage.

## Methods

### Study population

We evaluated a retrospective cohort in a single center, investigating 241 consecutive patients with stable CAD and myocardial damage admitted to Kagoshima University Hospital between January 2015 and August 2018 for PCI. All patients underwent coronary angiography and successful revascularization for the culprit lesion by PCI using a standard technique with second-generation drug-eluting stents and intravascular ultrasound. All patients were administered dual antiplatelet therapy (aspirin and thienopyridine: prasugrel or clopidogrel) before the procedure. Patients were followed up at our hospital or by their physician. In this study, patients with acute coronary syndrome were excluded. Patients who could not be tracked after discharge were also excluded. Acute coronary syndrome was defined as either acute myocardial infarction (ST-segment elevation myocardial infarction or non-ST segment elevation myocardial infarction) or unstable angina pectoris.

### Measurements and assessments of GNRI

Laboratory values were obtained at the time of admission before PCI. Levels of serum albumin, cholinesterase, high-sensitivity C-reactive protein (hs-CRP), high-density lipoprotein cholesterol (HDL-C), low-density lipoprotein cholesterol (LDL-C), total cholesterol (T-CHO), triglycerides, creatinine, uric acid, and fasting plasma glucose were measured, and estimated glomerular filtration rate (eGFR) was calculated using the Modification of Diet in Renal Disease equation with coefficients modified for Japanese patients as follows: eGFR (ml/min/1.73 m^2^) = 194 × serum creatinine (mg/dL)^−1.094^ × age (years)^−0.287^ (× 0.739 for female subjects) [11]. Blood samples were drawn after 12 h of fasting and serum levels of albumin were measured by modified bromocresol purple method (KAINOS, Tokyo, Japan).

This study assessed nutritional status using the GNRI, calculated using the following equation: GNRI = 14.89 × serum albumin level in g/dL + 41.7 × (body weight in kilograms/ideal body weight) [7]. Body weight/ideal body weight was set to 1 when the bodyweight of the patient exceeded the ideal bodyweight. Ideal bodyweight was calculated using a BMI of 22 kg/m^2^. BMI was calculated as bodyweight in kilograms divided by height in meters squared. Japan Society for the Study of Obesity suggests that ideal BMI is 22 kg/m^2^ and obesity is a BMI greater than or equal to 25 [12]. Therefore, we used 22 kg/m^2^ as ideal BMI.

### Definitions

In all patients, echocardiography was performed on admission before PCI, and left ventricular asynergy due to myocardial ischemia or infarction was defined as myocardial damage in this study. Conventional transthoracic echocardiographic examinations were performed using a commercially available ultrasound transducer and equipment. Echocardiography measurements were performed according to the guidelines of the American Society of Echocardiography [13]. We estimated left ventricular ejection fraction (LVEF) using the biplane Simpson method. Myocardial ischemia was evaluated by fractional flow reserve or myocardial perfusion single-photon emission computed tomography. Patients with GNRI < 92 at baseline were defined as the malnourished group based on previously published thresholds [7]. Hypertension was defined based on the following criteria: systolic blood pressure ≥ 140 mmHg, diastolic blood pressure ≥ 90 mmHg, or the use of antihypertensive medication. Diabetes mellitus was defined based on the following criteria: use of antihyperglycemic medication, fasting plasma glucose concentration > 126 mg/dL, or glycosylated hemoglobin concentration ≥ 6.5% (in accordance with the National Glycohemoglobin Standardization Program) [14]. Dyslipidemia was defined as low-density lipoprotein cholesterol ≥ 140 mg/dL, triglycerides ≥ 150 mg/dL, high-density lipoprotein cholesterol < 40 mg/dL, or use of antidyslipidemic medication. Current smokers were defined as those who were actively smoking at the time of admission.

### Clinical outcomes

Clinical outcomes were retrospectively collected during follow-up. All-cause death was defined as any death after PCI. Major cardiovascular and cerebrovascular events (MACCE) constituted a composite endpoint including all-cause death, non-fatal myocardial infarction, and ischemic stroke.

Patients were divided into a malnourished group (GNRI < 92) and a non-malnourished group, then MACCE after PCI was compared between groups.

### Statistical analysis

Quantitative data are presented as mean ± standard deviation or median and interquartile range (IQR). Fisher’s exact test was used to compare the incidences of categorical variables, expressed as frequency and percentage. Continuous variables were compared between malnourished and non-malnourished groups using Student’s t-test (for values showing a normal distribution) or the Wilcoxon rank-sum test (for values showing a non-normal distribution). Cumulative survival rate and rate of MACCE were estimated using a Kaplan–Meier curve evaluated by log-rank testing. Cox proportional hazards regression analysis was used to analyze factors associated with MACCE, reporting hazard ratios (HRs) and 95% confidence intervals (CIs). Variables showing values of *p* < 0.05 on univariate analysis were entered into multivariate analysis. Furthermore, Cox proportional hazards regression model was performed to assess HRs for MACCE, and results were expressed by forest plots. In addition, we conducted a test for interaction of malnutrition and hemodialysis. Values of *p* < 0.05 were considered to indicate statistical significance. Statistical analyses were performed using SAS software (JMP version 14.0).

## Results

### Baseline characteristics

The baseline clinical characteristics of patients are shown in Table 1. Mean age was 70 ± 11 years, and 163 patients (68%) were male. Mean GNRI was 100 ± 13, and participants comprised 55 malnourished patients (23%; GNRI < 92) and 186 non-malnourished patients (77%). Malnourished patients were older (77 ± 9 years) than those without malnutrition (67 ± 11 years, *p* < 0.001), and sex (male) had a significant difference between malnourished and non-malnourished patients (*p* = 0.009). The malnourished group showed a lower frequency of dyslipidemia and a higher frequency of hemodialysis compared with the non-malnourished group (*p* < 0.001 and *p* = 0.009, respectively). Median concentration of hs-CRP was higher in the malnourished group (3.1 mg/dL; IQR, 0.36–8.26 mg/dL) than in the non-malnourished group (0.19 mg/dL; IQR, 0.08–0.58 mg/dL; *p* < 0.001). Levels of T-CHO and triglycerides were lower in the malnourished group than in the non-malnourished group (*p* = 0.002 and *p* < 0.001, respectively) (Table 1).

**Table 1:** Baseline characteristics of study patients according to nutritional status

Variables	Overall	Malnourished group	Non-malnourished group	*p* value
	(n = 241)	(n = 55)	(n = 186)	
Age, years	70 ± 11	77 ± 9	67 ± 11	< 0.001
Sex, male, n (%)	163 (68)	29 (53)	134 (72)	0.009
BMI, kg/m^2^	23.1 [21.2, 25.5]	20.1 [18.5, 21.5]	24.5 [22.3, 26.4]	< 0.001
Risk factors, n (%)				
Hypertension	191 (79)	44 (80)	147 (79)	1.00
Diabetes mellitus	149 (61)	38 (69)	111 (60)	0.27
Dyslipidemia	164 (68)	26 (47)	138 (74)	< 0.001
Current smoking	45 (19)	7 (13)	38 (20)	0.24
Hemodialysis	66 (27)	23 (42)	43 (23)	0.009
Prior myocardial infarction	21 (9)	8 (15)	13 (7)	0.101
Prior stroke	15 (6)	2 (4)	13 (7)	0.53
Prior heart failure	20 (8)	7 (13)	13 (7)	0.18
Medication, n (%)				
Calcium-channel blocker	120 (50)	27 (49)	93 (50)	1.00
ACEI	19 (8)	4 (7)	15 (8)	1.00
ARB	79 (33)	14 (25)	65 (35)	0.25
β-blocker	48 (20)	10 (18)	38 (20)	0.85
Statin	112 (46)	18 (33)	94 (51)	0.022
Proton pump inhibitor	50 (21)	12 (22)	38 (20)	0.85
Laboratory data				
Cholinesterase, U/L	268 [208, 335]	194 [148, 253]	284 [236, 351]	< 0.001
hs-CRP, mg/dL	0.26 [0.09, 1.23]	3.1 [0.36, 8.26]	0.19 [0.08, 0.58]	< 0.001
T-CHO, mg/dL	164 [140, 197]	155 [131, 171]	172 [142, 203]	0.002
LDL-C, mg/dL	90 [71, 117]	86 [71, 112]	92 [71, 120]	0.22
HDL-C, mg/dL	46 [39, 58]	43 [35, 58]	46 [40, 57]	0.121
Triglyceride, mg/dL	107 [78, 155]	89 [64, 127]	114 [86, 159]	< 0.001
Albumin, g/dL	3.9 [3.4, 4.2]	3.0 [2.7, 3.4]	4.0 [3.7, 4.3]	< 0.001
Uric acid, mg/dL	5.5 [4.2, 6.7]	5.6 [3.8, 6.7]	5.4 [4.3, 6.7]	0.61
FPG, mg/dL	121 [98,153]	120 [99, 163]	121 [98, 151]	0.99
eGFR, mL/min/1.73 m^2^	47.9 [11.3, 65.6]	19.2 [9.5, 54.7]	51.4 [20.0, 70.7]	0.004
LVEF, %	55.0 [41.4, 66.2]	49.4 [38.8, 64.9]	56.8 [45.8, 67.2]	0.030

Values are shown as mean ± standard deviation or median with interquartile range

*ACEI* angiotensin-converting enzyme inhibitor, *ARB* angiotensin II receptor blocker, *BMI* body mass index, *eGFR* estimated glomerular filtration rate, *FPG* fasting plasma glucose, *HDL-C* high-density lipoprotein cholesterol, *hs-CRP* high-sensitivity C-reactive protein, *LVEF* left ventricular ejection fraction, *LDL-C* low-density lipoprotein cholesterol, *T-CHO* total cholesterol

### Clinical outcomes

**Fig. 1:**
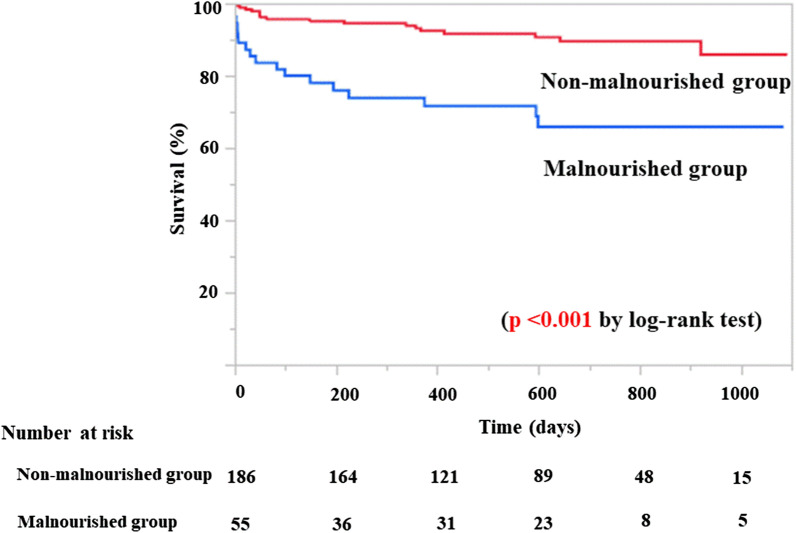
Kaplan–Meier analysis of survival rate based on nutritional status

**Fig. 2:**
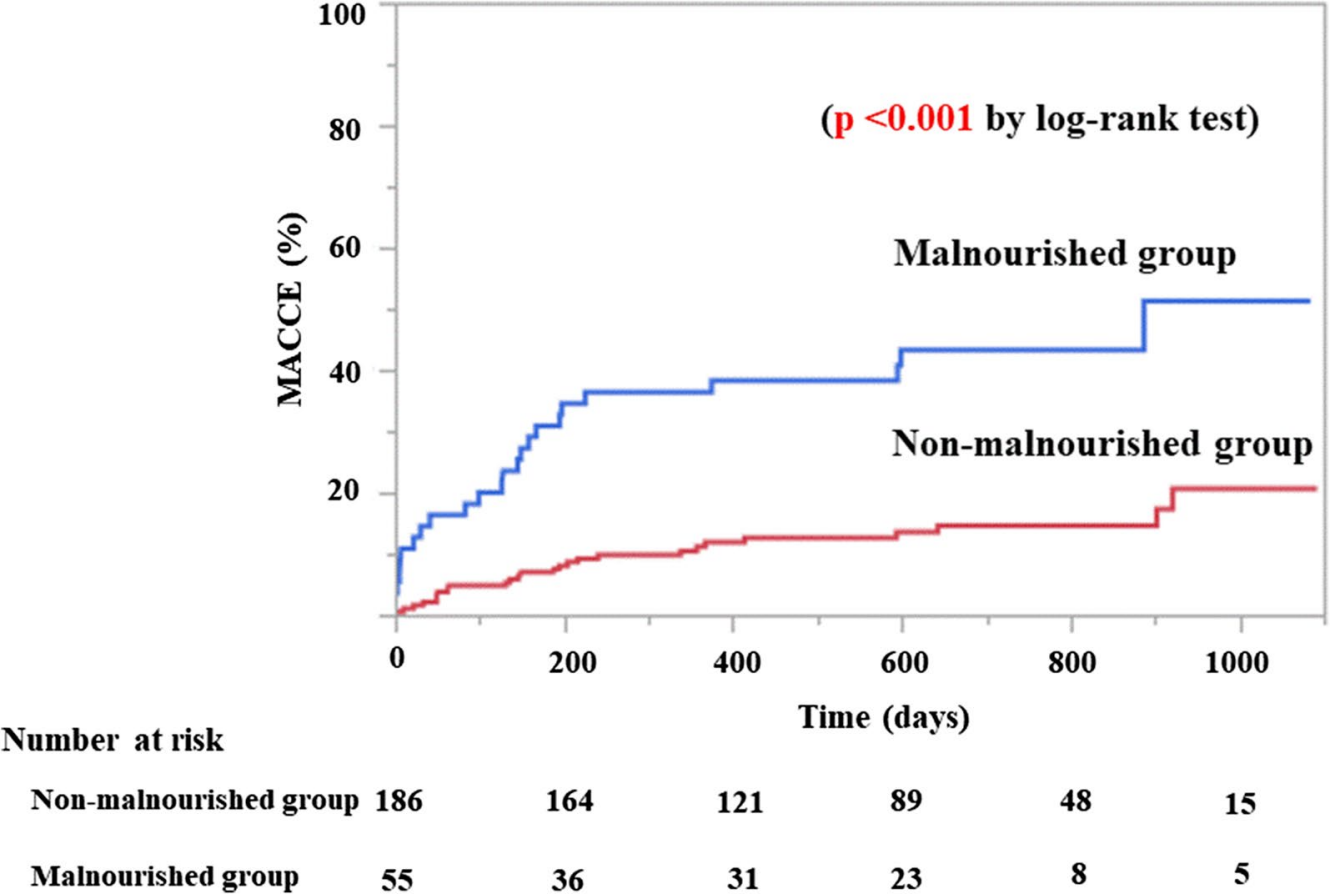
Cumulative incidences of MACCE based on nutritional status. *MACCE* major cardiovascular and cerebrovascular events

Mean duration of follow-up was 546 ± 310 days, with a maximum follow-up of 1092 days. Thirty-four patients (14%) died after PCI, and the frequency of all-cause death was significantly higher in the malnourished group (31%) than in the non-malnourished group (9%; *p* < 0.001). MACCE occurred more frequently in the malnourished group (38%) than in the non-malnourished group (11%; *p* < 0.001) (Table 2). Kaplan–Meier analysis showed a significantly lower survival rate after PCI in the malnourished group than in the non-malnourished group (*p* < 0.001) (Fig. 1). Cumulative incidence of MACCE including death was significantly higher in the malnourished group than in the non-malnourished group (*p* < 0.001) (Fig. 2).

**Table 2:** Long-term clinical outcome

	Overall	Malnourished group	Non-malnourished group	*p* value
	(n = 241)	(n = 55)	(n = 186)	
MACCE	42 (17)	21 (38)	21 (11)	< 0.001
Death	34 (14)	17 (31)	17 (9)	< 0.001
Non-fatal myocardial infarction	2 (1)	0 (0)	2 (1)	0.99
Ischemic stroke	6 (2)	4 (7)	2 (1)	0.025

Values are n (%)

*MACCE* major cardiovascular and cerebrovascular events

### Associations of baseline systemic factors with MACCE

**Fig. 3:**
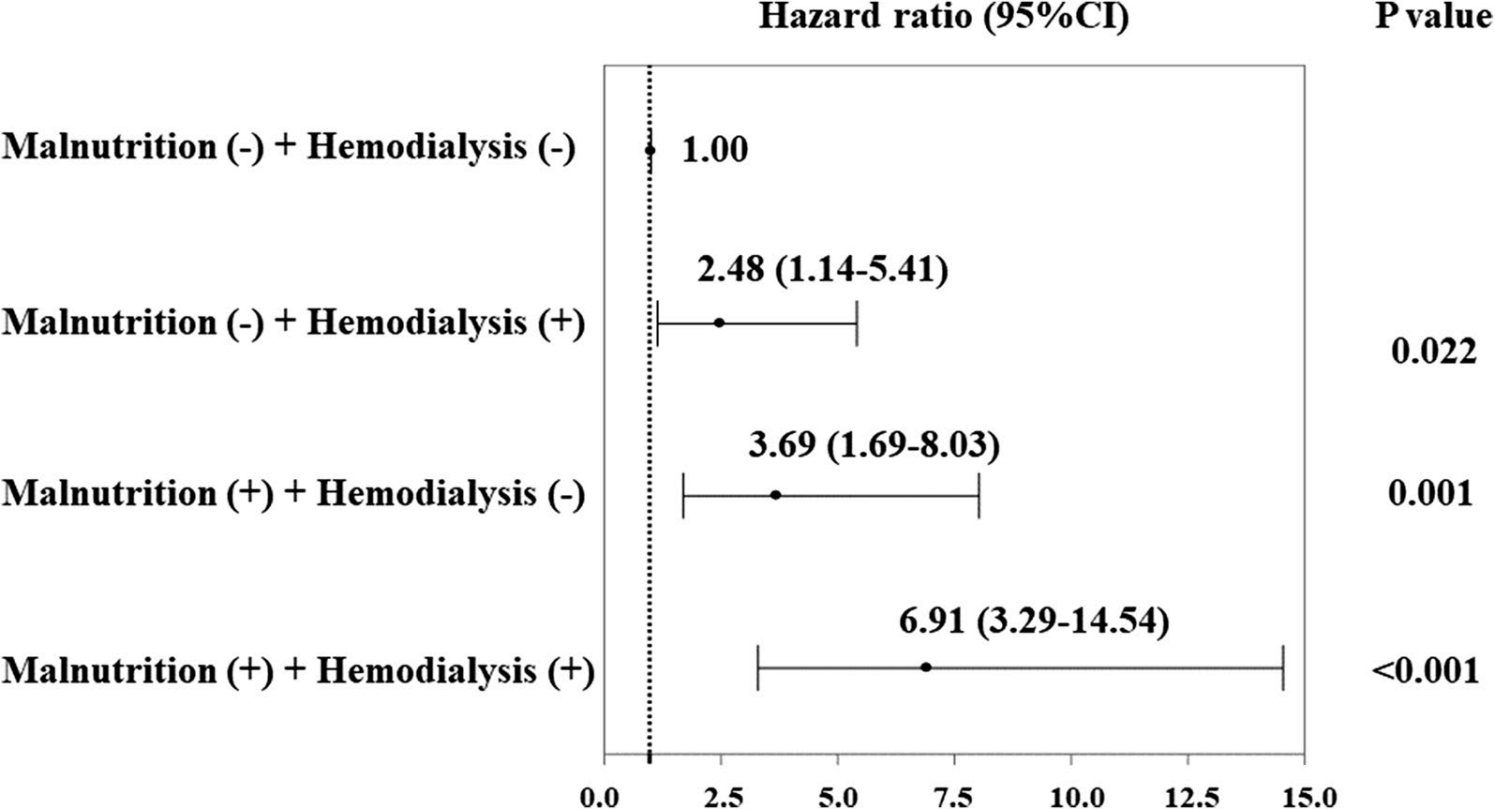
Cox proportional hazards models for MACCE. The forest plot shows the relative risk (with 95% confidence interval) of MACCE. *MACCE* major cardiovascular and cerebrovascular events

Cox proportional hazards regression analysis was performed to investigate associations between baseline characteristics including malnutrition and MACCE. In univariate analysis, age, BMI (HR 0.91; 95% CI 0.84–0.98; *p* = 0.016), malnutrition (HR 3.69; 95% CI 2.11–6.42; *p* < 0.001), hemodialysis (HR 2.63; 95% CI 1.51–4.58; *p* < 0.001), prior heart failure, cholinesterase, hs-CRP, albumin (HR 0.43; 95% CI 0.28–0.65; *p* < 0.001), eGFR, and LVEF were significantly associated with MACCE (Table 3). BMI and albumin were used to calculate GNRI. The eGFR was synonymous with hemodialysis, and the 95%CIs of cholinesterase and LVEF included 1.00. These factors were therefore not incorporated into the multivariate analysis. Multivariate analysis showed malnutrition (HR 2.30; 95% CI 1.13–4.67; *p* = 0.020) and hemodialysis (HR 2.17; 95% CI 1.19–3.93; *p* = 0.011) correlated positively with MACCE (Table 3). Furthermore, Cox proportional hazards models using malnutrition and hemodialysis revealed that patients with both malnutrition and hemodialysis showed greater risk of MACCE after PCI as compared to patients with neither malnutrition nor hemodialysis (HR 6.91; 95% CI 3.29–14.54; *p* < 0.001) (Fig. 3). A comparison of p-value for interaction of malnutrition and hemodialysis was *p* = 0.36.

**Table 3:** Cox proportional hazards regression analysis for MACCE

	Univariate analysis			Multivariate analysis		
	HR	(95% CI)	*p* Value	HR	(95% CI)	*p* Value
Age	1.04	(1.04–1.07)	0.004	1.03	(0.99–1.06)	0.092
Sex: male	1.11	(0.62–2.36)	0.58			
BMI	0.91	(0.84–0.98)	0.016			
Malnutrition (GNRI < 92)	3.69	(2.11–6.42)	< 0.001	2.30	(1.13–4.67)	0.020
Hypertension	1.21	(0.62–3.84)	0.34			
Diabetes mellitus	1.01	(0.59–1.78)	0.98			
Dyslipidemia	1.48	(0.84–2.61)	0.174			
Current smoking	1.42	(0.41–4.88)	0.57			
Hemodialysis	2.63	(1.51–4.58)	< 0.001	2.17	(1.19–3.93)	0.011
Prior myocardial infarction	1.64	(0.73–3.64)	0.22			
Prior stroke	3.16	(0.43–22.9)	0.26			
Prior heart failure	2.35	(1.10–5.01)	0.027	1.73	(0.78–3.85)	0.178
Calcium-channel blocker	1.03	(0.59–1.80)	0.91			
ACEI	0.67	(0.21–2.18)	0.52			
ARB	0.90	(0.63–1.30)	0.60			
β-blocker	0.82	(0.39–1.69)	0.59			
Statin	0.68	(0.38–1.20)	0.186			
Proton pump inhibitor	1.15	(0.59–2.25)	0.67			
Cholinesterase	0.99	(0.98–1.00)	< 0.001			
hs-CRP	1.08	(1.03–1.11)	< 0.001	1.01	(0.99–1.06)	0.52
T-CHO	1.00	(0.98–1.00)	0.056			
LDL-C	0.99	(0.97–1.01)	0.161			
HDL-C	0.98	(0.97–1.00)	0.28			
Triglycerides	0.99	(0.98–1.00)	0.073			
Albumin	0.43	(0.28–0.65)	< 0.001			
Uric acid	1.01	(0.91–1.05)	0.83			
FPG	1.00	(0.99–1.00)	0.61			
eGFR	0.98	(0.97–0.99)	< 0.001			
LVEF	0.97	(0.96–1.00)	0.010			

*CI* confidence interval, *HR* hazard ratio. Other abbreviations are as in Tables 1 and 2

## Discussion

In this retrospective cohort study of stable CAD patients with myocardial damage, we showed that: (1) patients with malnutrition displayed a higher incidence of MACCE as compared to patients without malnutrition; (2) malnutrition and hemodialysis were independent risk factors for MACCE in stable CAD patients with myocardial damage; and (3) patients with both malnutrition and hemodialysis were associated with an additive increase in the risk of MACCE.

Nutritional status is an important factor that affects the prognosis of various diseases [15, 16]. As a tool of assessment for nutritional status, serum albumin level and BMI are often used in routine clinical practice. However, both are influenced by various non-nutritional factors, including inflammation, fluid status, and liver and renal functions [17–19]. BMI or albumin alone may thus be insufficient for assessing nutritional status. GNRI, as an index combining BMI and albumin [7], has recently been widely used in simple screenings of nutritional status and may be useful to overcome the shortcomings of individual indicators such as serum albumin and BMI. Recent studies have revealed that low GNRI is associated with worsened prognosis in patients with cardiovascular disease [8, 9, 20], and Kunimura et al. [21] demonstrated that low GNRI was independently associated with poor cardiac outcomes in patients with preserved ejection fraction after elective PCI for stable CAD. The present study demonstrated that malnutrition as assessed by GNRI represented an independent risk factor for MACCE in stable CAD patients with myocardial damage. Stable CAD patients with myocardial damage show poor prognosis compared to those without myocardial damage [10]. In addition, the ICELAND MI study demonstrated that patients with unrecognized myocardial infarction were at higher risk of all-cause mortality than patients without myocardial infarction. Furthermore, among patients with first myocardial infarction, previously unrecognized myocardial infarction was independently related to poor long-term clinical outcome, with a more than threefold risk of mortality [22]. Intensive care is therefore needed to prevent recurrent cardiovascular events in CAD patients with myocardial damage compared to those without myocardial damage, and total management including prevention of energy and protein waste and feeding energy based on nutritional status may improve the prognosis for stable CAD patients with myocardial damage.

In the present study, hemodialysis was also independently associated with MACCE. Chronic kidney disease has been established as a risk factor for cardiovascular morbidity and mortality [23]. The ISCHEMIA-CKD trial showed stable CAD patients with advanced kidney disease including hemodialysis had high incidences of death and cardiovascular events compared to those without advanced kidney disease [24, 25]. Those previous reports may support the results of the present study. In patients with end-stage renal disease, malnutrition has been thought to have a close relationship with inflammation and atherosclerosis, and the concept of malnutrition-inflammation-atherosclerosis (MIA) syndrome has been suggested [26, 27]. MIA syndrome was then recognized to be an important clinical issue which should be addressed in patients with end-stage renal disease. Furthermore, previous studies have suggested that inflammation may deteriorate a generally catabolic state, leading to acceleration of protein degradation and suppression of protein synthesis in patients with end-stage renal disease [28]. These mechanisms may cause protein-energy malnutrition [29]. Although nutritional status is affected by the interaction of multiple other factors, inflammation is recognized as a central factor in malnutrition. Chronic inflammation is known to lead to increase oxidative stress and the development of severe endothelial dysfunction, in turn leading to cardiovascular disease [27]. Indeed, we showed that patients with malnutrition and hemodialysis had additively elevated incidences of MACCE in this study. Statin therapy has not been found to achieve any obvious effect on prevention of cardiovascular events in patients with advanced kidney disease [30]. Hence, a novel strategy for preventing cardiovascular events may be needed in patients with advanced kidney disease. In recent years, some anti-inflammatory therapies that do not affect lipid levels have demonstrated the ability to reduce cardiovascular events in CAD patients [31, 32]. Multidisciplinary care including nutritional management and the aforementioned anti-inflammatory therapies may have the possibility of stopping the malignant cycle of MIA syndrome in patients with end-stage renal disease.

This study showed several limitations. First, the present retrospective study involved a relatively small cohort of patients. Second, although we assessed nutritional status using the GNRI as a combined index of BMI and albumin, we could not completely exclude systemic illnesses such as occult malignancies affecting BMI or albumin. In addition, the present study assessed the GNRI only on admission and did not assess changes in GNRI. Third, we were unable to evaluate another malnutrition index such as the Nutritional Risk Index, Mini Nutritional Assessment Short-Form scale or Maastricht Index, because the present study is retrospective, and we do not have the data those indexes need. Fourth, participants comprised 66 patients with hemodialysis (27%) in this study, and it is reported that hemoglobin A1c may be underestimated in patients with hemodialysis. Thus, hemoglobin A1c was not included in the analysis. Fifth, the association of malnutrition with CAD or gender is an important issue to investigate; however, we were unable to address this association in the present study. Further clinical investigations are necessary to clarify this association.

## Conclusions

The current study revealed malnutrition as assessed by GNRI offered an independent risk factor for MACCE among stable CAD patients with myocardial damage. Assessment of nutritional status may be helpful for stratifying the risk of cardiovascular events and encouraging improvements in nutritional status.

## Data Availability

The datasets used and/or analyzed during the current study are available from the corresponding author on reasonable request.

## Abbreviations

ACEI: Angiotensin-converting enzyme inhibitor
ARB: Angiotensin II receptor blocker
BMI: Body mass index
CAD: Coronary artery disease
CI: Confidence interval
eGFR: Estimated glomerular filtration rate
FPG: Fasting plasma glucose
GNRI: Geriatric nutritional risk index
HDL-C: High-density lipoprotein cholesterol
HR: Hazard ratio
hs-CRP: High-sensitivity C-reactive protein
IQR: Interquartile range
LVEF: Left ventricular ejection fraction
LDL-C: Low-density lipoprotein cholesterol
MACCE: Major cardiovascular and cerebrovascular events
MIA syndrome: Malnutrition-inflammation-atherosclerosis syndrome
PCI: Percutaneous coronary intervention
T-CHO: Total cholesterol
